# The Australasian Resuscitation In Sepsis Evaluation: Fluids or vasopressors in emergency department sepsis (ARISE FLUIDS), a multi‐centre observational study describing current practice in Australia and New Zealand

**DOI:** 10.1111/1742-6723.13469

**Published:** 2020-02-10

**Authors:** Gerben Keijzers, Stephen PJ Macdonald, Andrew A Udy, Glenn Arendts, Michael Bailey, Rinaldo Bellomo, Gabriel E Blecher, Jonathon Burcham, Andrew R Coggins, Anthony Delaney, Daniel M Fatovich, John F Fraser, Amanda Harley, Peter Jones, Frances B Kinnear, Katya May, Sandra Peake, David McD Taylor, Patricia Williams, the ARISE FLUIDS Observational Study Group, Khanh Nguyen, Lai Heng Foong, Carolyn Hullick, Richard McNulty, Andrew Na, Christopher Trethewy, Lucy Lutze, Michael Zhang, Tim Cowan, Paul Middleton, Suzanne Avis, Sam Vidler, Mark Salter, Simon Janes, Anthony Delaney, Tom Harwood, Matthew Oliver, Farzad Jazayeri, Sarah Jones, Michael Davoren, Andrew Coggins, Bibhu Pradhananga, Peter Jones, Lynette Newby, Sierra Beck, Brad Sandleback, Sophie Rabas, Simon Harger, Eunicia Tan, Rima Song, Marc Gutenstein, Andrew Munro, Michael Connely, Jennifer Goodson, Alastair Mclean, Christine Brabyn, Saptarshi Mukerji, Harnah Simmonds, Paul Young, Yulia Sugeng, Cheryl Bird, Amanda McConnell, Gerben Keijzers, Peter Henderson, David Johnson, Siegfried Perez, Abbas Mahani, Ulrich Orda, Ogilvie Thom, Kym Roberts, Frances Kinnear, Sarah Hazelwood, Hanh Pham, Rob Eley, Georgia Livesay, Michael Devlin, Ian Murdoch, Erik Wood, Julian Williams, Nathan Brown, Alex King, Jan Sadewasser, Leonie Jones, Vinay Gangathimmaiah, Anit Manudhane, Daniel Haustead, Juan‐Carlos Ascencio‐Lane, David McD. Taylor, Paul Buntine, Katie Walker, Pourya Pouryahya, Daniel Crompton, Ron Sultana, Timothy Campbell, Rosamond Dwyer, Gabriel Blecher, Jonathan Knott, Biswadev Mitra, Carl Luckhoff, Russel Young, Natalie Rudling, Ashes Mukherjee, Kerry‐Lee Dyke, Casey Parker, Glenn Arendts, Alannah Cooper, Yusuf Nagree, Keng Koay, Coert Kruger, Nicole Ghedina, Ben Smedley, Jonathon Burcham, Stephen Macdonald, Helen Hamersley

**Affiliations:** ^1^ Department of Emergency Medicine Gold Coast University Hospital Gold Coast Queensland Australia; ^2^ School of Medicine Bond University Gold Coast Queensland Australia; ^3^ School of Medicine Griffith University Gold Coast Queensland Australia; ^4^ Centre for Clinical Research in Emergency Medicine Harry Perkins Institute of Medical Research Perth Western Australia, Australia; ^5^ Emergency Department, Royal Perth Hospital The University of Western Australia Perth Western Australia, Australia; ^6^ Australian and New Zealand Intensive Care Research Centre School of Public Health and Preventive Medicine, Monash University Melbourne Victoria Australia; ^7^ Department of Intensive Care and Hyperbaric Medicine The Alfred Melbourne Victoria Australia; ^8^ School of Medicine The University of Western Australia Perth Western Australia Australia; ^9^ Department of Medicine and Radiology The University of Melbourne Melbourne Victoria Australia; ^10^ Department of Intensive Care Austin Hospital Melbourne Victoria Australia; ^11^ School of Medicine The University of Melbourne Melbourne Victoria Australia; ^12^ Emergency Department Monash Medical Centre, Monash Health Melbourne Victoria Australia; ^13^ Monash Emergency Research Collaborative, School of Clinical Sciences at Monash Health Monash University Melbourne Victoria Australia; ^14^ Emergency Medicine and Trauma Westmead Hospital Sydney New South Wales Australia; ^15^ Malcolm Fisher Department of Intensive Care Medicine Royal North Shore Hospital Sydney New South Wales Australia; ^16^ Northern Clinical School, Sydney Medical School The University of Sydney Sydney New South Wales Australia; ^17^ Division of Critical Care and Trauma The George Institute for Global Health, The University of New South Wales Sydney New South Wales Australia; ^18^ Faculty of Medicine The University of Queensland Brisbane Queensland Australia; ^19^ Critical Care Research Group The Prince Charles Hospital Brisbane Queensland Australia; ^20^ Intensive Care Unit St Andrew's War Memorial Hospital Brisbane Queensland Australia; ^21^ Critical Care Management Team Queensland Children's Hospital Brisbane Queensland Australia; ^22^ The University of Queensland Brisbane Queensland Australia; ^23^ School of Medicine, The University of Auckland, Auckland New Zealand; ^24^ Adult Emergency Department Auckland City Hospital Auckland New Zealand; ^25^ Emergency and Children's Services The Prince Charles Hospital Brisbane Queensland Australia; ^26^ Department of Intensive Care Medicine The Queen Elizabeth Hospital Adelaide South Australia Australia; ^27^ Faculty of Health and Medical Sciences, School of Medicine Adelaide University Adelaide South Australia Australia; ^28^ School of Epidemiology and Preventive Medicine Monash University Melbourne Victoria Australia; ^29^ Emergency Medicine Research Austin Hospital Melbourne Victoria Australia; ^30^ Department of Medicine The University of Melbourne Melbourne Victoria Australia

**Keywords:** emergency department, fluid therapy, hypotension, sepsis, vasopressor

## Abstract

**Objectives:**

To describe haemodynamic resuscitation practices in ED patients with suspected sepsis and hypotension.

**Methods:**

This was a prospective, multicentre, observational study conducted in 70 hospitals in Australia and New Zealand between September 2018 and January 2019. Consecutive adults presenting to the ED during a 30‐day period at each site, with suspected sepsis and hypotension (systolic blood pressure <100 mmHg) despite at least 1000 mL fluid resuscitation, were eligible. Data included baseline demographics, clinical and laboratory variables and intravenous fluid volume administered, vasopressor administration at baseline and 6‐ and 24‐h post‐enrolment, time to antimicrobial administration, intensive care admission, organ support and in‐hospital mortality.

**Results:**

A total of 4477 patients were screened and 591 were included with a mean (standard deviation) age of 62 (19) years, Acute Physiology and Chronic Health Evaluation II score 15.2 (6.6) and a median (interquartile range) systolic blood pressure of 94 mmHg (87–100). Median time to first intravenous antimicrobials was 77 min (42–148). A vasopressor infusion was commenced within 24 h in 177 (30.2%) patients, with noradrenaline the most frequently used (*n* = 138, 78%). A median of 2000 mL (1500–3000) of intravenous fluids was administered prior to commencing vasopressors. The total volume of fluid administered from pre‐enrolment to 24 h was 4200 mL (3000–5661), with a range from 1000 to 12 200 mL. Two hundred and eighteen patients (37.1%) were admitted to an intensive care unit. Overall in‐hospital mortality was 6.2% (95% confidence interval 4.4–8.5%).

**Conclusion:**

Current resuscitation practice in patients with sepsis and hypotension varies widely and occupies the spectrum between a restricted volume/earlier vasopressor and liberal fluid/later vasopressor strategy.

**Key findings**

- The optimal volume and timing of fluid resuscitation and initiation of vasopressor support in patients with sepsis and hypotension is unknown.
- Current resuscitation practices in patients with sepsis and hypotension vary widely in our study, with fluid volume given in the first 24 hours ranging from 1–12 L.
- This study will inform the design of a randomised controlled trial comparing a restricted volume (earlier vasopressor) to a liberal fluid (later vasopressor) strategy.

## Introduction

Hypotension in sepsis results from a variable combination of fluid extravasation, peripheral vasodilatation and myocardial depression.^1^ A cornerstone of immediate management is the administration of intravenous (IV) fluid, followed by commencement of a vasopressor infusion if hypotension and/or poor end‐organ perfusion persists. In the absence of high‐quality evidence to support specific fluid volumes, a 30 mL/kg or greater IV fluid bolus is recommended by the Surviving Sepsis Campaign.^2^ However, the evaluation of a protocolised sepsis care bundle in almost 50 000 patients did not find any association between administration of 30 mL/kg IV fluid within 3 h of ED presentation, and differential mortality.^3^

Despite the almost universal clinical acceptance of fluid administration in sepsis, there are multiple studies that suggest possible harm from the liberal use of this intervention.^4^, ^5^, ^6^, ^7^, ^8^, ^9^, ^10^ As these studies were conducted in intensive care settings,^4^, ^5^ conducted in low‐income countries,^6^, ^7^, ^8^ or involved preclinical models,^10^ their findings cannot be generalised to adults presenting to the ED with suspected sepsis in Australia and New Zealand. Indeed, an alternative approach to sepsis resuscitation is earlier initiation of vasopressor infusions, with observational studies reporting increased mortality when this is delayed.^11^, ^12^ Moreover, a recent single centre double‐blind randomised controlled trial conducted in a tertiary hospital in Thailand demonstrated that administration of low‐dose noradrenaline resulted in greater shock resolution by 6 h, as compared with placebo (75 *vs* 48%).^13^

Accordingly, the optimal volume and timing of fluid resuscitation and initiation of vasopressor support in patients with sepsis and hypotension represent significant knowledge gaps.^14^, ^15^, ^16^ A comprehensive understanding of current resuscitation practices is essential for the design and conduct of future large‐scale clinical trials evaluating the effects of alternative strategies (such as restricted fluid therapy combined with early vasopressor use), in patients presenting to the ED with septic shock.

As such, the aim of the Australasian resuscitation in sepsis evaluation: Fluid or vasopressors in ED sepsis (ARISE FLUIDS) observational study was to describe current resuscitation practices and outcomes in patients presenting to the ED with sepsis, specifically to determine: (i) current IV fluid and vasopressor administration; (ii) in‐hospital mortality and receipt of organ support; and (iii) the incidence of patients presenting to the ED with sepsis and hypotension.

## Methods

### *Design, setting and participants*

The methodology of the ARISE FLUIDS observational study has been published previously,^17^ and was endorsed by the Australasian College for Emergency Medicine Clinical Trials Network. Human Research and Ethics and governance approval was obtained for all sites according to local requirement and data collection with a waiver of patient consent was approved. Briefly, ARISE FLUIDS was a prospective, multi‐centre observational study conducted in 70 EDs in Australia and New Zealand, where individual sites could select a consecutive 30‐day data collection period commencing between 13 September 2018 and 15 December 2018, with final data collected on 13 January 2019. During the site‐selected data collection period, adult patients presenting to the ED were eligible for inclusion if they met the following inclusion criteria: (i) clinically suspected infection; (ii) IV antimicrobials commenced; and (iii) systolic blood pressure (SBP) <100 mmHg at any time in ED despite at least 1000 mL IV fluid resuscitation. This had to be given as fluid bolus(es) of at least 500 mL, within 60 min per bolus, inclusive of pre‐hospital fluids. Exclusion criteria were: (i) hypotension suspected to be due to another cause e.g. arrhythmia, haemorrhage; (ii) confirmed or suspected pregnancy; (iii) comorbidities such that intensive care unit (ICU) or high dependency unit admission for vasopressor use is not appropriate; (iv) death deemed imminent or inevitable by the treating clinician; (v) life expectancy <90 days due to an underlying illness; and (vi) transfer from another acute care hospital.

### *Screening, data collection and follow‐up*

**Figure 1:**
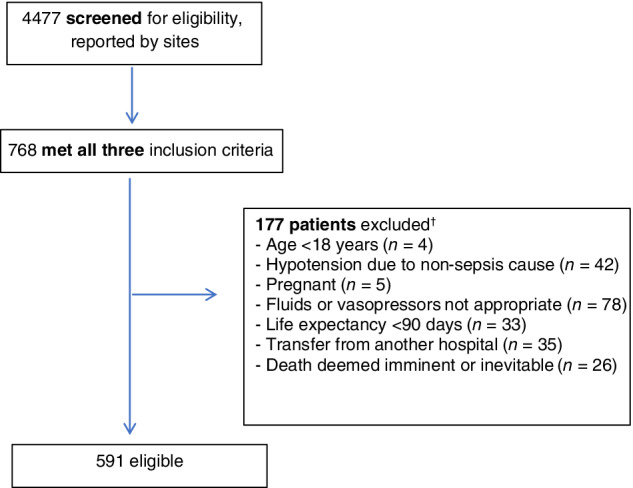
Patient flowchart. †Some patients met >1 exclusion criterion.

All sites were provided with a preformatted screening form and standardised education material to optimise screening, identification of eligible patients and data collection. For patients meeting all study entry criteria (Fig. 1), a detailed case report form was completed.^17^ Relevant variables and outcomes included: (i) baseline demographics and comorbidities; (ii) vital signs and laboratory variables at baseline and at 6 h post‐enrolment; (iii) type, timing and dose of IV fluid and vasopressor administration from ED presentation up to 24 h post‐enrolment (including fluids administered pre‐hospital); (iv) time to commencing the first IV antimicrobial agent; (v) source of sepsis and source control; (vi) ED duration of stay; (vii) ICU/high dependency unit admission; and (viii) in‐hospital mortality. For patients admitted to the ICU, we collected additional information on ICU duration of stay and receipt and duration of invasive mechanical ventilation, vasopressors and acute renal replacement therapy. De‐identified data were entered by local site investigators or the coordinating site into a purpose‐built web‐based database (REDCap®) hosted by the Australian and New Zealand Intensive Care Research Centre, Monash University. The ARISE FLUIDS study conforms to the STROBE principles for reporting of observational studies.^18^

**Figure 2:**
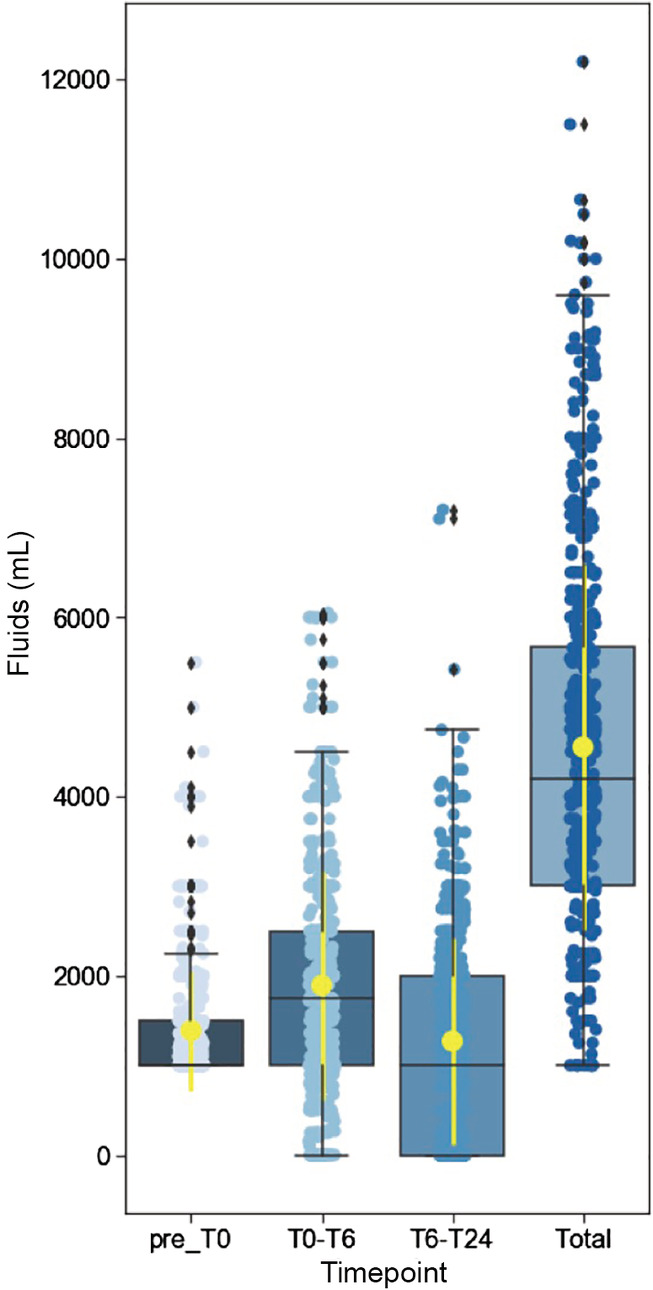
Intravenous fluid volumes administered in all eligible patients (*n* = 591). Box plots with median and IQR shown. Yellow: mean and standard deviation.

### *Statistical analysis*

We did not perform a formal sample size calculation as this was a descriptive study. The analysis plan anticipated data collection on 400 patients.^17^ Continuous variables are reported as mean and standard deviation (SD) or median and interquartile range (IQR) and categorical variables as proportions (%) as appropriate. Baseline characteristics, process‐of‐care measures (e.g. fluid volume at 6‐ and 24‐h post‐enrolment and frequency, timing and duration of vasopressor administration) and outcomes (e.g. ED disposition, ICU admission, receipt and duration of organ support and in‐hospital mortality) are reported.

Subgroups based on pre‐enrolment characteristics were specified *a priori*: (i) hospital type (rural/regional, metropolitan/district, private, tertiary); (ii) age <65 *versus* ≥65 years; (iii) quartiles of the Acute Physiology and Chronic Health Evaluation (APACHE II) score at T0; (iv) presence of cardiovascular disease; (v) SBP <90 *versus* ≥90 mmHg; (vi) lactate <2.0 *versus* ≥2.0 mmol/L; (vii) SBP <90 mmHg and lactate ≥2.0 mmol/L and first dose of IV antimicrobials commenced patients *versus* patients not meeting these criteria; (vi) source of sepsis; abdominal source *versus* other; respiratory source *versus* other; (vii) quartiles of fluid volume administered up to 6 h post‐enrolment; and (viii) quartiles of time to commencing a vasopressor infusion. Between‐group differences in in‐hospital mortality, ICU admission and receipt and duration of organ support were compared for the quartiles of volume of fluid administered and quartiles of time to commencing vasopressors sub‐groups. Chi‐squared or Fisher's exact test for categorical variables were used and Student's *t*‐test or Mann–Whitney *U*‐test used for continuous variables.

## Results

The 70 participating EDs had a combined adult patient attendance during their respective 30‐day data collection periods of 295 904 patients. Of 4477 patients screened, 591 (13.2%) patients met the eligibility criteria for detailed data collection (Fig. 1).

#### Baseline characteristics

Baseline characteristics are summarised in Table 1. The mean (SD) age was 62.4 (19.1) years and APACHE II score 15.2 (6.7). The median time from ED presentation to commencing IV antimicrobials was 77 (IQR 42–148) minutes. More than one‐third (37.6%, *n* = 222) had no prior co‐morbidities and 9.8% (*n* = 58) lived in a residential care setting. Table S1 portrays vital signs, blood gas analyses, receipt of invasive ventilation and laboratory data at baseline, 6, and 24 h.

**Table 1:** Baseline characteristics and comorbidities at T0 (*n* = 591)

Variable		*n*
Age, years, mean (SD)	62.4 (19.1)	583
Female sex, *n* (%)	290 (49.2)	590
Weight, kg, median (IQR)	75 (62.3–90)	363
Lactate, mmol/L, median (IQR)	2.2 (1.4–3.4)	474
APACHE II score, mean (SD)	15.2 (6.67)	590
Time from triage to T0, h, median (IQR)	2.43 (1.28–4.28)	590
Time from triage to first IV antimicrobials, min, median (IQR)	77 (42–148)	584
Total fluid volume prior to T0, mL, median (IQR)	1000 (1000–1500)	590
Invasive ventilation, *n* (%)	4 (0.86)	466
Non‐invasive ventilation, *n* (%)	17 (3.6)	567
Prior living status, *n* (%)		589
Home	526 (89.3)	
Residential care	58 (9.8)	
Comorbidities, *n* (%)		591
Respiratory disease	150 (25.4)	
Immuno‐suppressed	151 (25.6)	
Cardiac disease	190 (32.1)	
Liver disease	40 (6.8)	
Renal dialysis	27 (4.6)	
None	222 (37.6)	

T0 is defined as the time when all three inclusion criteria were met. Comorbidities as defined as per APACHE II definitions. Invasive ventilation indicates mechanical ventilation. APACHE II, Acute Physiology and Chronic Health Evaluation.

#### Fluid resuscitation and vasopressor therapy

Table 2 and Figure 2 summarise fluid and vasopressor administration up to 24 h post‐enrolment (including pre‐hospital fluids) for the overall cohort and the SBP and lactate subgroups. Overall, the fluid volume administered in the first 6 h post‐enrolment was 1789 mL (1000–2500) and, from pre‐enrolment to 24 h post‐enrolment, 4200 mL (3000–5661). Patients with a baseline SBP <90 mmHg, lactate ≥2 mmol/L or both, received total median fluid volumes of 4582, 4645 and 4805 mL up to 24 h, respectively. The majority of resuscitation fluid in the first 24 h consisted of 0.9% saline and balanced isotonic fluid (Table S2).

**Table 2:** Fluid volume and vasopressor use, for overall group and selected subgroups

	Overall (*n* = 591)	SBP <90 (*n* = 191)	Lactate ≥2 (*n* = 264)	SBP <90 and lactate ≥2 (*n* = 82)
Total fluid administered, mL				
Pre‐T0 h				
Median (IQR)	1000 (1000–1500)	1000 (1000–1500)	1000 (1000–1900)	1000 (1000–1900)
Mean (SD)	1389 (647)	1377 (642)	1461 (669)	1486 (753)
Between T0 and T6 h				
Median (IQR)	1789 (1000–2500)	2000 (1250–3000)	2000 (1000–3000)	2015 (1320–300)
Mean (SD)	1908 (1230)	2254 (1252)	2126 (1244)	2317 (1231)
Between T6 and T24 h				
Median (IQR)	1000 (200–2000)	1200 (500–2000)	1165 (540–2000)	1250 (625–2000)
Mean (SD)	1273 (1111)	1383 (1085)	1394 (1111)	1354 (1031)
Total: preT0‐T24 h				
Median (IQR)	4200 (3000–5661)	4582 (3500–6200)	4645 (3498–6108)	4805 (3900–6170)
Mean (SD)	4518 (1980)	4962 (1986)	4936 (1973)	5124 (1883)
Vasopressors started in ED, *n* (%)	134 (22.7)	74 (38.7)	84 (31.8)	42 (51.2)
Total fluid volume prior starting vasopressor infusion, mL				
Median (IQR)	2000 (1500–3000)	2000 (1500–3000)	2000 (1500–3000)	2000 (1250–3000)
Mean (SD)	2465 (1280)	2255 (1258)	2461 (1292)	2297 (1394)
Vasopressor infusion started before T24, *n* (%)	177 (30.2)	92 (48.4)	114 (43.3)	53 (64.6)
Time from Triage to start of vasopressor infusion, h, median (IQR)	4.7 (2.7–7.8)	4.2 (2.2–7.0)	3.8 (2.3–6.7)	3.5 (2.1–6.3)
Time from T0 to start of vasopressor infusion, h, median (IQR)	2.5 (0.8–5.0)	2.0 (0.6–4.5)	1.9 (0.7–4.5)	1.8 (0.4–3.1)
Duration of vasopressor infusion, h, median (IQR)	26.2 (12–48)	31 (15.6–52)	29.8 (15–59)	32.1 (19–69)
Type and median duration of individual vasopressors				
Noradrenaline, *n* (%)	138 (78)	70 (76.1)	95 (83.3)	43 (81.1)
Duration, h, median (IQR)	5.27 (3.0–9.2)	31 (17–50)	33 (14–54)	39 (19.5–55)
Metaraminol, *n* (%)	74 (42)	38 (41.8)	45 (39.5)	20 (37.7)
Duration, h, median (IQR)	5.8 (2.7–26)	7 (2.4–26)	4 (2.4–17.1)	4 (2.4–17.2)
Adrenaline, *n* (%)	15 (8.6)	11 (12.2)	11 (9.8)	7 (13.5)
Duration, h, median (IQR)	4.6 (2.0–13.8)	11 (5.5–22.7)	13.7 (3.3–32)	19.6 (5.5–42)
Vasopressin, *n* (%)	19 (10.9)	10 (11.1)	18 (16.1)	7 (13.5)
Duration, h, median (IQR)	10.8 (5.4–15.1)	22.7 (17–29)	21.2 (16–36)	19.7 (16–23)
Dobutamine, *n* (%)	4 (2.3)	2 (2.2)	3 (2.7)	1 (1.9)
Duration, h, median (IQR)	9.1 (3.6–33.5)	N/A	N/A	N/A
CVC or PICC inserted before T24 hrs	126 (21.5)	65 (34.2)	94 (35.7)	41 (50.0)
Time from Triage to CVC/PICC insertion, hrs, median (IQR)	6.5 (3.5–10.1)	5.9 (3.5–8.9)	6.1 (3.0–8.9)	6.2 (3.5–8.0)

Fluid volumes did not include maintenance fluids. T0 is defined as the time when all three inclusion criteria were met. CVC, central venous catheter; PICC, peripherally inserted central catheter.

Almost one‐third of patients (30.2%, *n* = 177) received a vasopressor infusion in the first 24 h post‐enrolment, with noradrenaline (78%, *n* = 138) and metaraminol (42%, *n* = 74) most frequently used (Table 3). In patients with a SBP <90 mmHg at baseline, 48.4% (*n* = 92) received a vasopressor infusion up to 24 h post‐enrolment and in patients with a SBP <90 mmHg and a lactate ≥2 mmoL/L, almost two‐thirds (64.6%, *n* = 53) received a vasopressor. A central venous catheter or peripherally inserted central catheter were inserted in the first 24 h in 126 patients (21.5%).

**Table 3:** Outcomes. Source of sepsis, ED disposition, ICU outcomes and organ support and mortality, for overall group and selected subgroups

	Overall (*n* = 581)	SBP <90 (*n* = 191)	Lactate ≥2 (*n* = 264)	SBP <90 and lactate ≥2 (*n* = 82)
Source of sepsis, *n* (%)				
Respiratory	198 (33.7)	64 (33.5)	88 (33.3)	25 (30.5)
Urinary	147 (25.0)	45 (23.6)	58 (22)	20 (24.4)
Skin/soft tissue	67 (11.4)	28 (14.7)	29 (11)	11 (13.4)
Bloodstream	14 (2.4)	4 (2.1)	7 (2.7)	3 (3.7)
Abdo/pelvis	80 (13.6)	24 (12.6)	43 (16.3)	10 (12.2)
Central nervous system	6 (1)	0 (0)	2 (0.8)	0 (0)
Bone/joint	7 (1.2)	3 (1.6)	4 (1.5)	1 (1.2)
Other	9 (1.5)	2 (1)	2 (0.8)	1 (1.2)
No source found	60 (10.2)	20 (10.5)	29 (11)	10 (12.2)
Source control procedures performed, *n* (%)	50 (8.5)	21 (11.1)	9.1 (24)	10 (12.2)
Discharge destination from ED, *n* (%)				
ICU	172 (29.4)	84 (44.4)	109 (41.6)	46 (57.5)
HDU	30 (5.1)	13 (6.9)	14 (5.3)	5 (6.3)
CCU	14 (2.4)	0 (0)	2 (0.8)	0 (0)
Ward	314 (53.7)	74 (39.2)	115 (43.9)	21 (26.3)
ED short stay ward	11 (1.9)	3 (1.6)	1 (0.4)	0 (0)
Operating theatre	13 (2.2)	6 (3.2)	8 (3.1)	2 (2.5)
Transfer to other hospital	31 (5.3)	9 (4.8)	11 (4.2)	4 (5.0)
ED length of stay, h, median (IQR)	7.9 (5.35–13.4)	7.3 (4.8–11)	7.7 (5.1–11.5)	6.5 (4.3–9.8)
ICU outcomes				
Admitted to ICU within 24 h, *n* (%)	218 (37.1)	107 (56.6)	132 (50.0)	54 (65.9)
ICU duration of stay, days, median (IQR)	2.4 (1.3–4.6)	2.4 (1.2–4.5)	2.7 (1.5–4.6)	2.7 (1.7–4.5)
Patients receiving invasive ventilation, *n* (%)	36 (16.7)	14 (13.3)	27 (20.8)	8 (15.1)
Duration of ventilation, days, median (IQR)	5.6 (2.0–7.08)	4.35 (1.8–7.5)	5.5 (1.9–7.3)	4.4 (1.9–7.7)
Patients receiving acute (RRT), *n* (%)	13 (6.1)	7 (6.7)	8 (6.3)	3 (5.8)
Duration of RRT, days, median (IQR)	2.2 (0.6–8.1)	4.0 (0.2–6.8)	3.1 (0.7–8.8)	4.0 (0.2–6.8)
Hospital outcomes				
Hospital length of stay, days, median (IQR)	5.1 (2.8–10.1)	6.2 (3.6–12)	7.1 (4.0–15)	8.4 (4.0–15)
Discharge destination for survivors, *n* (%)				
Home	451 (79.8)	137 (75.7)	202 (80.2)	62 (80.5)
Care facility	79 (14)	27 (14.9)	29 (11.5)	9 (11.7)
Mortality, *n* (%)				
ED	4 (0.7)	2 (1)	2 (0.8)	2 (2.4)
ICU	18 (8.6)	8 (7.8)	12 (9.4)	1 (2)
In‐hospital	36 (6.2)	17 (9.3)	21 (8.2)	6 (7.8)

ICU outcomes uses number of patients admitted to ICU as denominator. HDU, high dependency unit; RRT, renal replacement therapy.

#### Clinical outcomes

Overall, one‐third (33.7%, *n* = 198) of patients had a respiratory source and a quarter (25.0%, *n* = 147) had a urinary source, with no source found in 10.2% (*n* = 60). The median ED length of stay was 7.9 h (5.4–13.4) and over one‐third (37.1%, *n* = 218) of patients were admitted to an intensive care setting in the first 24 h. ICU admission rates were higher for the subgroups SBP <90 mmHg (56.6%, *n* = 107) and SBP <90 mmHg and lactate ≥2 mmol/L (65.9%, *n* = 54). For patients admitted to ICU within 24 h, the median length of stay was 2.4 days (1.3–4.6). One in six (16.7%, *n* = 36) patients admitted to ICU received invasive ventilation and 6.1% (*n* = 13) received acute renal replacement therapy. Hospital duration of stay for the whole patient cohort was 5.1 (2.8–10.1) days with an in‐hospital mortality of 6.2% (95% confidence interval 4.5–8.5%).

#### Subgroup analyses by fluid volume and time to vasopressor infusion

Pre‐specified sub‐group analyses are provided in Tables S3–S11. Tables 4, 5 show the clinical outcomes according to the volume of fluid administered in the 6 h post‐enrolment and the timing of commencing a vasopressor infusion.

**Table 4:** Baseline characteristics, fluid volume, need for organ support and mortality; overall group and by quartiles of fluid administered T0–T6

	Overall (*n* = 584)	0–1000 mL (*n* = 188)†	1001–1800 mL (*n* = 104)	1801–2501 mL (*n* = 147)	>2501 mL (*n* = 145)
Female, *n* (%)	286 (49)	87 (46.3)	49 (47.1)	78 (53.1)	72 (49.7)
Age, years	62.5 (19.1)	65.8 (19)	61.3 (16.6)	61.6 (18.6)	59.8 (19.2)
Lactate, mmol/L	2.2 (1.4–3.4)	1.8 (1.3–3.1)	2.1 (1.3–3.4)	2.2 (1.5–3.4)	2.8 (1.8–4.1)
APACHE II score	15.2 (6.7)	14.4 (6.4)	14.5 (6.3)	15.2 (6.5)	16.6 (7.3)
SBP, mmHg	94.8 (13.7)	97.4 (12.5)	94.3 (11)	94.5 (14)	91.8 (16.1)
Time from triage to antibiotics, mins	77 (42–147)	85 (46–172)	97 (41–180)	77 (47–135)	59 (34–117)
Fluid volume administered, mL					
Pre‐T0 h					
Median (IQR)	1000 (1000–1500)	1000 (1000–1535)	1000 (1000–1500)	1000 (1000–1500)	1000 (1000–2000)
Mean (SD)	1389 (647)	1362 (571)	1410 (743)	1330 (564)	1442 (678)
Between T0 and T6 h					
Median (IQR)	1789 (1000–2500)	900 (469–1000)	1400 (1250–1538)	2000 (2000–2306)	3200 (3000–4000)
Mean (SD)	1908 (1230)	689 (380)	1404 (212)	2141 (211)	3612 (871)
Between T6 and T24 h					
Median (IQR)	1000 (200–2000)	1000 (0–1825)	1250 (759–1883)	1000 (0–2000)	1550 (850–2370)
Mean (SD)	1273 (1111)	975 (1091)	1369 (993)	1275 (1121)	1590 (1119)
Total: pre‐T0 and T24 h					
Median (IQR)	4200 (3000–5661)	3000 (2000–4000)	4000 (3300–4792)	4500 (3753–5675)	5350 (3600–6250)
Mean (SD)	4543 (1969)	3011 (1348)	4183 (1269)	4729 (1316)	6600 (1724)
Total fluid volume administered prior to starting vasopressors, mL, *n*	134	23	22	37	50
Median (IQR)	2000 (1500–3000)	1750 (1125–3000)	2000 (1500–2500)	2000 (1850–3800)	2750 (2000–3500)
Mean (SD)	246 (1280)	1887 (981)	2072 (935)	2492 (1220)	2894 (1440)
Vasopressor infusion started in ED, *n* (%)	132 (22.6)	23 (12.2)	22 (21.1)	37 (25.2)	50 (34.5)
Vasopressor infusion started before T24, *n* (%)	176 (30.1)	36 (19.1)	27 (26)	44 (29.9)	69 (47.6)
Duration of vasopressor infusion, h, median (IQR)	26.6 (12–48)	24.5 (11–38)	21.7 (6–45)	17.5 (8–48)	36 (22–69)
Time to start vasopressor infusion from T0, h, median (IQR)	2.5 (0.9–5.0)	2.4 (1.2–4.7)	2.1 (0.5–5.4)	2.1 (0.7–5.5)	2.7 (1.0–5.3)
ICU outcomes					
Admitted to ICU within 24 h, *n* (%)	217 (37.2)	45 (23.9)	32 (30.8)	53 (36.1)	87 (60.0)
Patients receiving invasive ventilation, *n* (%)	36 (16.7)	4 (9.1)	4 (12.5)	9 (17.0)	19 (22.1)
Duration of ventilation, days, median (IQR)	5.6 (2.0–7.1)	4.4 (1.4–6.2)	7.8 (0.6–8.4)	6.0 (5.7–7.5)	4.1 (2.0–6.9)
Patients receiving RRT, *n* (%)	13 (6.1)	2 (4.7)	1 (3.1)	3 (5.7)	7 (8.2)
Duration of RRT, days, median (IQR)	2.2 (0.6–6.8)	0.9 (0.5–1.3)	4.1 (4.1–4.1)	0.6 (0.2–11)	4.0 (0.9–20)
ICU mortality, *n* (%)	18 (8.6)	6 (14.3)	4 (12.5)	5 (9.6)	3 (3.6)
Hospital mortality, *n* (%)	36 (6.3)	12 (6.5)	7 (6.7)	8 (5.5)	9 (6.4)
Hospital length of stay, days, median (IQR)	5.1 (2.8–10)	4.5 (2.5–8.2)	4.3 (2.8–8.3)	5.8 (6.1–15)	7.0 (3.7–14)

† As 88 patients had exactly 1000 mL administered in the first 6 h, the first and second fluid quartile do not have the same number of patients.

APACHE II: Acute Physiology and Chronic Health Evaluation; RRT, renal replacement therapy; SBP, systolic blood pressure; T0, time when all three inclusion criteria were met.

**Table 5:** Baseline characteristics, fluid volume, need for organ support and mortality; overall group and by quartiles of time to start of vasopressor infusion

	Overall (*n* = 177)	0–2.7 h (*n* = 45)	2.71–4.7 h (*n* = 44)	4.71–7.7 h (*n* = 44)	>7.71 h (*n* = 44)
Female, *n* (%)	78 (44.1)	20 (44.4)	19 (43.2)	21 (47.7)	18 (40.9)
Age, years, mean (SD)	65.2 (16.3)	68.6 (12.8)	65.5 (18.4)	68.9 (15.3)	58.3 (16.4)
Lactate, mmol/L, median (IQR)	3.0 (2.0–4.9)	3.7 (2.4–6.3)	2.7 (2.1–4.5)	3.3 (1.7–4.7)	2.8 (1.6–4)
APACHE II score, mean (SD)	17.8 (6.3)	20.5 (6.2)	18 (6.1)	18.6 (5.7)	14.1 (5.0)
SBP, mmHg, mean (SD)	89 (13.7)	84.3 (12.3)	91.3 (17.6)	88.2 (8.7)	92.5 (12.1)
Time from triage to antibiotics, mins, median (IQR)	59 (33–118)	43 (20–71)	53 (27–100)	67 (42–130)	88 (46–196)
Fluid volume administered, mL					
Pre‐T0 h					
Median (IQR)	1250 (1000–2000)	1000 (1000–1650)	1283 (1000–2000)	1475 (1000–2000)	1383 (1000–2000)
Mean (SD)	1533 (6980)	1488 (748)	1533 (678)	1552 (625)	1561 (755)
Between T0 and T6 h					
Median (IQR)	2188 (1250–3250)	2260 (1320–3750)	2088 (1188–3475)	2263 (1250–3000)	2102 (1295–3100)
Mean (SD)	2420 (1465)	2582 (1560)	2324 (1431)	2300 (1315)	2466 (1567)
Between T6 and T24 h					
Median (IQR)	1710 (950–2500)	1592 (600–2677)	1200 (650–2200)	1120 (943–2000)	2000 (1780–2553)
Mean (SD)	1666 (1145)	1710 (1348)	1435 (1037)	1373 (999)	2134 (1038)
Total: pre‐T0–T24 h					
Median (IQR)	5300 (4000–7000)	5565 (3600–7350)	5000 (4030–6650)	5156 (3850–6208)	4653 (2750–6003)
Mean (SD)	5567 (2130)	5704 (2425)	5260 (1878)	5141 (2017)	6160 (2072)
Total fluid volume administered prior to starting vasopressors, mL, *n*	134	44	31	31	21
Median (IQR)	2000 (1500–3000)	2000 (1000–2625)	2000 (2000–3100)	2184 (1750–2854)	3500 (3000–4100)
Mean (SD)	2465 (1280)	2015 (1169)	2565 (1036)	2400 (1074)	3652 (1448)
Vasopressor infusion started in ED, *n* (%)	126 (71.2)	43 (95.6)	31 (70.5)	31 (70.5)	21 (47.7)
Vasopressor infusion started before T24, *n* (%)	177 (100)	45 (100)	44 (100)	44 (100)	44 (100)
Duration of vasopressor infusion, h, median (IQR)	26.6 (12–48)	32 (11–88)	26 (12–44)	22 (17–38)	31 (8.1–51)
Time to start vasopressor infusion from T0, h, median (IQR)	2.5 (0.8–5.0)	0.5 (0–1.25)	2.0 (1.1–2.7)	3.8 (2.2–5.0)	7.1 (4.9–9.5)
Admitted to ICU within 24 h, *n* (%)	155 (88.1)	42 (93.3)	37 (84.1)	38 (88.4)	38 (86.4)
Patients receiving invasive ventilation, *n* (%)	31 (20.3)	12 (28.6)	8 (22.2)	2 (5.3)	9 (24.3)
Duration of ventilation, days, median (IQR)	5.6 (5.7–6.5)	2.7 (1.8–7.1)	5.7 (1.8–7.5)	9.5 (5.7–13)	5.2 (2.9–32)
Patients receiving acute RRT, *n* (%)	13 (8.6)	3 (7.3)	5 (3.9)	2 (5.4)	3 (8.1)
Duration of RRT, days, median (IQR)	2.2 (0.6–6.8)	2.2 (0.2–4.0)	4.1 (0.9–6.8)	0.3 (0.2–0.5)	20 (1.3–23)
ICU mortality, *n* (%)	16 (10.7)	6 (14.6)	2 (5.7)	3 (8.1)	5 (13.9)
Hospital mortality, *n* (%)	22 (13.1)	6 (14)	4 (9.8)	7 (17.1)	5 (11.6)
Hospital length of stay, days, median (IQR)	8.6 (4.4–18)	9.3 (4.2–18)	7.8 (4.1–15)	7.1 (4.9–13)	9.8 (4.4–19)

ICU outcomes uses number of patients admitted to ICU as denominator.

APACHE II, Acute Physiology and Chronic Health Evaluation; RRT, renal replacement therapy; SBP, systolic blood pressure; T0, time when all three inclusion criteria were met.

The highest fluid quartile received more than 5 times the fluid volume as the lowest quartile in the first 6 h post‐enrolment (3612 *vs* 689 mL) and twice as much in the first 24 h (6600 *vs* 3011 mL). Between quartile 1 and quartile 4, there was an increase in vasopressor initiation (from 19.1 to 47.6%), ICU admission (from 23.9 to 60%) and receipt of invasive ventilation (from 9.1 to 22.1%) (Table 4). Duration of vasopressor therapy varied between 17.5 and 24.5 h in the lowest three fluid quartiles and was 36 (IQR 22–69) hours in the highest fluid quartile. Although in‐hospital mortality was similar across all fluid quartiles, duration of hospital stay was 4.5 days in quartile 1 and 7.0 days in quartile 4.

The quartile of patients who commenced a vasopressor infusion the earliest (within 2.7 h of ED presentation) tended to be older and tended to have a higher baseline lactate and APACHE II score as well as a lower SBP compared to the remaining quartiles (Table 5). The total volume of fluid administered between 0 and 6 h and up to 24 h was similar across the vasopressor quartiles, although the quartile of patients who received vasopressors the latest (after 7.71 h), were given most fluid (3500 mL [3000–4100]) prior to commencement of vasopressors. The in‐hospital mortality for patients receiving vasopressors was 13.1% (*n* = 23) with a median hospital length of stay of 8.6 (4.4–18) days.

## Discussion

#### Key findings

Our study provides critical contemporary data concerning sepsis resuscitation in EDs throughout Australia and New Zealand. Namely, patients received on average 2 L of IV fluid in the first 6 h after enrolment, and 4.5 L in the first 24 h in hospital (including pre‐enrolment). Approximately 30% required vasopressor support within the first 24 h, after a median of 2 L of IV fluid, and 4.7 h from ED triage. A SBP <90 mmHg and/or lactate ≥2 mmoL/L was associated with greater fluid administration, more frequent use of vasopressors, and higher rates of admission to ICU. Patients receiving larger volume fluid resuscitation more commonly needed vasopressors and mechanical ventilation, and had a longer hospital stay. Overall, in‐hospital mortality was low (6.2%).

#### Comparison with other studies

The routine care arms in the ARISE Early Goal Directed Therapy^19^ and Restricted Fluid Resuscitation in Sepsis associated Hypotension (REFRESH)^20^ randomised controlled trials delivered different fluid volumes from pre‐enrolment to 6 h post (4.2 *vs* 3 L). This may reflect differences in severity of disease or inclusion criteria, but could also suggest adoption of a more fluid restrictive approach over time. The routine care group in REFRESH received a median of 1715 mL between 0 and 6 h and 4250 mL from pre‐enrolment to 24 h, which is similar to the overall findings in the current study.

The quartile of patients receiving the lowest fluid volume (median 900 mL administered in the first 6 h after enrolment and 3 L in the first 24 h), had somewhat less fluid administered than the restricted volume arm of REFRESH.^20^ In contrast, the quartile of patients in our study receiving the most fluid were administered 3.2 L in the first 6 h after enrolment and 5.4 L from pre‐enrolment to 24 h, with the overall range of fluid given in the first 24 h varied between 1 and 12.2 L. These findings are consistent with sepsis being a highly variable clinical syndrome, where an individualised approach to fluid resuscitation is often employed.

Of note was the low in‐hospital mortality observed in the present study (6.2%, 95% confidence interval 4.5–8.5%), whereas other Australasian studies of patients with sepsis and hypotension have reported in‐hospital mortality rates between 18 and 25%.^21^, ^22^ In contrast, and consistent with our study, when assessing patients without limitations of care, the 30‐day mortality was 6.2% in a cohort of 399 ED patients with septic shock in a tertiary hospital in Australia.^21^ Temporal improvements of sepsis care and an associated decrease in mortality in Australia and New Zealand may also partially explain our findings.^22^

Our study used a similar methodology to that undertaken in 32 Australian and New Zealand hospitals conducted in 2009.^23^ However, the inclusion criteria for that study were a SBP <90 mmHg despite a 500 mL bolus or a lactate greater than 4 mmol/L. Notably, all patients with sepsis were included in the 2009 study, whereas in our study patients with limitations of care were excluded. These differences in eligibility may explain the higher APACHE II score in the 2009 study compared to ARISE FLUIDS (19 ± 8.2 *vs* 15.2 ± 6.7) as well as the higher mortality (23.1%). In ARISE 2009, approximately 2 L was administered between 0 and 6 h, which was similar to ARISE FLUIDS. Almost one‐third (32%) of patients received vasopressors in the first 6 h after enrolment in ARISE 2009, whereas in ARISE FLUIDS a similar proportion had vasopressors started before 24 h (30.2%). The median time to vasopressor commencement was 4.6 h (2.7–7.8) which was shorter than median time to central line access of 6.5 h (3.5–10.1), indicating vasopressor delivery through a peripheral line occurred. This practice is supported by an analysis of patients who received peripheral vasopressors in the ARISE Early Goal Directed Therapy trial^19^ which showed that this was associated with some improvements in processes of care, and not associated with differential mortality.^24^ A recent systematic review concluded when given for a limited duration and under close observation, adverse events of peripheral vasopressors are rare.^25^

#### Study implications

Our study found significant heterogeneity in fluid volume resuscitation in hypotensive patients with sepsis, implying that the clinical environment might potentially support a controlled trial in this area.^26^ With falling mortality among patients with sepsis who are eligible for ICU care, there is a growing recognition that long‐term quality of life among survivors relates to organ failure.^27^, ^28^ In this respect, our data suggest a potential relationship between greater fluid volume resuscitation in the first 6 and 24 h and organ dysfunction, implying that the need for invasive organ support represents a logical endpoint for future work. Finally, our study provided important insights into the yield of our screening procedures. One in every 66 adult ED presentations (1.51%) were screened with an enrolment rate of 13.2%, or a ‘number needed to screen’ of 7.6. This corresponded with 2 in every 1000 adult attendances in the present study being eligible, which is consistent with prior research.^29^

### *Limitations*

The participating sites were self‐selected based on an expression of interest via the Australasian College for Emergency Medicine, with data collection occurring only in spring and summer. However, the 70 hospitals represented a wide geographical spread ranging from tertiary to rural and remote facilities with varying levels of onsite ICU facilities. As such we believe our findings have robust external validity. Although in‐hospital mortality is lower in this study than most others performed in similar settings, comparisons are difficult to make, as compared to other studies we did not report 90‐day mortality. This was because we utilised an endpoint more proximal to the exposure of interest (e.g. fluid administration in ED), as 90‐day mortality is likely to be confounded by other factors. We excluded 26 patients where death was imminent and 33 with a life‐expectancy <90 days.

It is possible that not all patients were screened or enrolled. In particular, the low mortality rate raises the question as to whether sicker patients were missed. A more likely explanation is that patients who were not eligible for ICU admission were excluded from our study, particularly since we had dedicated site clinician–investigators actively screening for suitable patients. In the Australasian ED setting these patients not eligible for ICU admission represent a substantial proportion of patients who die as a result of their sepsis, with mortality rates varying between 47 and 66%.^22^, ^23^ Further, missing data were inevitable as patients received routine care which may not include all relevant variables. Any ‘missingness’ is therefore likely to be random.

The observation that an increasing volume of fluid was associated with a greater proportion requiring vasopressors and/or mechanical ventilation should be viewed cautiously. Although greater fluid administration has been associated with harm, this is clearly confounded by illness severity. In this respect, these data should be considered hypothesis‐generating at best, and reinforces the need for future systematic research in this area.

## Conclusion

The ARISE FLUIDS observational study is the largest Australasian observational study providing a 30‐day snapshot of contemporary ED practice across a wide range of settings, from rural/regional to metropolitan teaching hospitals. Current resuscitation practices in patients with sepsis and hypotension in Australia and New Zealand vary widely, occupying the spectrum between a restricted volume/earlier vasopressor and liberal fluid/later vasopressor strategy.

## Supporting information


**Table S1**. Vital signs and laboratory results at eligibility (T0) and 6 and 24 h.
**Table S2**. Main fluid types administered.
**Table S3**. Systolic blood pressure at T0; ≥90 *versus* <90 mmHg.
**Table S4**. Lactate at T0. <2 *versus* ≥2 mmol/L.
**Table S5**. Systolic blood pressure <90 and lactate ≥2 mmol/L at T0 *versus* patients not meeting these criteria.
**Table S6**. Abdominal source of sepsis *versus* other source.
**Table S7**. Respiratory source of sepsis *versus* other source.
**Table S8**. Age <65 *versus* ≥65 years.
**Table S9**. Cardiovascular comorbidities absent or present.

**Table S10**. Severity of illness – by APACHE II quartiles at T0.
**Table S11**. Hospital type.
