# Supplementary material for: The Australasian Resuscitation In Sepsis Evaluation: Fluids or vasopressors in emergency department sepsis (ARISE FLUIDS), a multi‐centre observational study describing current practice in Australia and New Zealand
Source: Emerg Med Australas. 2020 Feb 10;32(4):586–98. doi: 10.1111/1742-6723.13469 (PMC7496107; doi:10.1111/1742-6723.13469)
Supplement: Supplementary file 1 — Table S1. Vital signs and laboratory results at eligibility (T0) and 6 and 24 h. Table S2. Main fluid types administered. Table S3. Systolic blood pressure at T0; ≥90 versus <90 mmHg. Table S4. Lactate at T0. <2 versus ≥2 mmol/L. Table S5. Systolic blood pressure <90 and lactate ≥2 mmol/L at T0 versus patients not meeting these criteria. Table S6. Abdominal source of sepsis versus other source. Table S7. Respiratory source of sepsis versus other source. Table S8. Age <65 versus ≥65 years. Table S9. Cardiovascular comorbidities absent or present. [file EMM-32-586-s001.docx]

**Supplemental tables**

| **Variables** | **T0** | *N* | **T6** | *N* | **T24** | *N* |
| --- | --- | --- | --- | --- | --- | --- |
| Temperature, ^o^C | 37.5 (1.2) | *582* | 36.9 (0.9) | *468* | 36.9 (0.8) | *460* |
| Heart rate, beats/min | 97 [84-112] | *589* | 89 (19) | *516* | 86 (16.8) | *475* |
| SBP, mmHg | 94 [87-100] | *587* | 105 (16.4) | *515* | 112 (15.9) | *474* |
| DBP, mmHg | 56 [50-63] | *585* | 60 (11.1) | *515* | 63 (10.6) | *473* |
| Respiratory rate, breaths/min | 22 (6.3) | *587* | 19 [17-22] | *504* | 18 [16-20] | *461* |
| SpO_2,_ % | 95.7 (3.4) | *589* | 96.1 (2.8) | *512* | 96.2 (2.5) | *476* |
| Supplemental oxygen, n (%) | 213 (36.5%) | *584* | NA |  | NA |  |
| FiO_2_, % | NA |  | 0.21 [0.21-0.28] | *489* | 0.21 [0.21-0.28] | *457* |
| GCS, total | 14.5 (1.5) | *575* | 14.5 (2.0) | *399* | 14.5 (1.9) | *344* |
| Blood gas performed, n (%) | 467 (79.4%) | *588* | 176 (30%) | *587* | 143 (24.4%) | *586* |
| PH | 7.40 [7.35-7.44] | *467* | 7.35 (0.1) | *173* | 7.37 (0.08) | *142* |
| PCO_2,_ mmHg | 40.9 (10.6) | *464* | 36.5 (10.5) | *174* | 37.5 (10.1) | *141* |
| HCO_3,_ mmol/L | 23.9 (4.9) | *467* | 20 (5.5) | *172* | 21.4 (5.0) | *143* |
| Lactate, mmol/L | 2.1 [1.4-3.4] | *462* | 1.8 [1.1-2.9] | *175* | 1.5 [1.0-2.1] | *139* |
| *Laboratory results*  Sodium, mmol/L  Potassium, mmol/L  Urea, mmol/L  Creatinine, μmol/L  Chloride, mmol/L  Bilirubin, μmol/L  Albumin, g/L  Glucose, mmol/L  Haemoglobin, g/L  Haematocrit, %  White Cell Count, 10^9^/L  Platelets, 10^9^/L  INR | 136 [133-139]  4.06 (0.70)  7.4 [5-11.7]  98 [72-150]  99.8 (6.1)  14 [9-22]  32 [28-37]  6.6 [5.6-8.1]  125 (21.7)  38 (6.4)  13.0 (7.4)  224 (113)  1.2 [1.1-1.4] | *568*  *555*  *540*  *564*  *527*  *494*  *507*  *506*  *566*  *536*  *563*  *551*  *201* | 136 (5.7)  4.1 (0.69)  8.4 [5.5-13.3]  108 [75-166]  105 (7.5)  15 [10-25]  26.3 (5.2)  7 [5.5-8]  112 (21.8)  33.7 (7.3)  13.6 (7.2)  188 (115)  1.3 [1.2-1.5] | *133*  *133*  *98*  *101*  *120*  *71*  *80*  *97*  *120*  *84*  *88*  *86*  *45* | 137 (4.2)  4.0 (0.56)  6.6 [4.1-12.4]  78.5 [63-121]  105 (6.1)  11 [8-22]  25.7 (5.1)  7.4 (3.2)  111(17.2)  33.6 (5.0)  12.4 (8.0)  198 (112)  1.7 (0.8) | *218*  *218*  *176*  *190*  *194*  *134*  *152*  *127*  *206*  *175*  *183*  *180*  *65* |

**Table A**. *Vital signs and laboratory results at eligibility (T0) and 6 and 24 hrs*

SBP Systolic Blood Pressure, DBP Diastolic Blood Pressure, data in mean (SD), median [IQR] or n (%), INR: International Normalised Ratio, NA: not available

**Supplemental Table B.** Main fluid types administered

| *Fluid Type administered, mL* | **Overall**  ***N=581*** | ***SBP<90***  ***N=191*** | **lactate≥2**  ***N=264*** | **SBP<90 and lactate≥2**  ***N=82*** |
| --- | --- | --- | --- | --- |
| **0.9% Saline, mL**  **T0-T6**  Median [IQR]  Mean (SD)  Range  **T6-T24**  Median [IQR]  Mean (SD)  Range  **Balanced Isotonic, mL**  **T0-T6**  Median [IQR]  Mean (SD)  Range  **T6-T24**  Median [IQR]  Mean (SD)  Range  **Albumin, mL,**  **T0-T6**  Median [IQR]  Mean (SD)  Range  **T6-T24**  Median [IQR]  Mean (SD)  Range | 1000 [250-2000]  1234 (1060)  0-5000  125 [0-10000]  633 (821)  0-4000  0 [0-1000]  623 (921)  0-5000  0 [0-1000}  497 (787)  0-4000  0 [0-0]  46 (172)  0-1000  0 [0-0]  68 (240)  0-2000 | 1166 [982-2000]  1485 (1088)  0-5000  100 [0-1000]  584 (796)  0-4000  0 [0-1000]  700 (995)  0-5000  0 [0-1000]  602 (852)  0-4000  0 [0-0]  55 (173)  0-1000  0 [0-0]  84 (256)  0-1592 | 1000 [500-2000]  1316 (1082)  0-4000  63 [0-1000]  571 (765)  0-3100  250 [0-1000]  745 (1004)  0-5000  0 [0-1000]  587 (816)  0-3500  0 [0-0]  55 (180)  0-1000  0 [0-0]  114 (322)  0-2000 | 1375 [785-2260]  1517 (1088)  0-4000  0 [0-1000]  549 (759)  0-3000  0 [0-1000]  709 (1091)  0-5000  0 [0-1000]  592 (724)  0-3000  0 [0-0]  68 (191)  0-800  0 [0-0]  143 (353)  0-1592 |

SBP: systolic blood pressure; SD: standard deviation; IQR: interquartile range

**Supplemental table C.** Systolic Blood Pressure at T0; ≥90 vs <90 mmHg

|  | **Overall**  ***N=587*** | ***SBP* ≥*90***  ***N=396*** | **SBP<90**  **N=191** | **p-value** |
| --- | --- | --- | --- | --- |
| Female, n (%)  Age, years  Lactate T0, mmol/L  APACHE II score  SBP at T0, mmHg  Time from triage to antibiotics, mins | 289 (49.2%)  62.4 (19.1)  2.2 [1.4-3.4]  15.2 (6.7)  94.7 (13.7)  77 [42-149] | 198 (50.0%)  61.3 (19.4)  2.0 [1.3-3.1]  14.2 (6.5)  101 (11.2)  88 [45-156] | 91 (47.6%)  65.1 (18.1)  2.4 [1.6-3.6]  17.4 (6.4)  81.3 (7.0)  69 [38-129] | 0.59  0.025  0.001  <0.001  <0.001  0.028 |
| *Fluid volume administered, mL*  Pre-T0,  Between T0-T6  Between T6-T24  Total: preT0-T24 | 1000 [1000-1500]  1898 (1226)  1000 [225-2000]  4510 (1980) | 1000 [1000-1500]  1727 (1176)  1000 [0-2000]  4291 (1942) | 1000 [1000-1500]  2254 (1252)  1200 [500-2000]  4962 (1986) | 0.41  <0.0001  0.07  <0.0001 |
| Total fluid volume administered prior to starting vasopressors, mL | *N=133*  2000 [1500-3000] | *N=58*  2500 [2000-4000] | *N=74*  2000 [1500-3000] | 0.013 |
| Vasopressor infusion started in ED, n (%)  Vasopressor infusion started before T24, n (%)  Duration of vasopressor infusion, hrs  Time to start vasopressor infusion from T0, hrs | 132 (22.5%)  174 (29.8%)  27.2 [12-51] | 58 (14.6%)  82 (20.8%)  23 [8.2-48] | 74 (38.7%)  92 (48.4%)  31 [16-52] | <0.001  <0.001  <0.001 |
| *ICU Outcomes*  Admitted to ICU within 24 hours, n (%) Patients receiving invasive ventilation, n (%) Duration of ventilation, days Patients receiving RRT, n (%)  Duration of RRT, days | 215 (36.8%)  35 (16.5%)  5.6 [2.0-7.3]  12 (5.7%)  3.1 [0.5-8.8] | 108 (27.3%)  21 (19.6%)  6.1 [2.7-7.1]  5 (4.7%)  2.2 [1.3-11] | 107 (56.6%)  14 (13.3%)  4.4 [1.8-7.5]  7 (6.7%)  4.0 [0.2-6.8] | <0.001  0.22  0.56  0.53  0.75 |
| ICU mortality, n (%)  Hospital Mortality, n (%)  Hospital LOS, days | 17 (8.2%)  35 (6.1%)  5.1 [2.8-10] | 9 (8.6%)  18 (4.6%)  4.9 [2.7-8.8] | 8 (7.8%)  17 (9.3%)  6.2 [3.2-12.6] | 0.85  0.028  0.004 |

T0= time when all 3 inclusion criteria were met; APACHE II: Acute Physiology and Chronic Health Evaluation; SBP: Systolic Blood Pressure; ED: emergency department; ICU: intensive care unit; CCU: Coronary Care Unit; RRT: renal replacement therapy; LOS: Length of stay

For all supplemental tables: ICU outcomes uses number of patients admitted to ICU as denominator. Some variables had missing data accounting for proportions reported.

**Supplemental table D.** Lactate at T0. <2 vs ≥2 mmol/L

|  | **Overall**  ***N=474*** | ***Lactate<2***  ***N=210*** | **Lactate ≥2**  **N=264** | **p-value** |
| --- | --- | --- | --- | --- |
| Female, n (%)  Age, years  Lactate T0, mmol/L  APACHE II score  SBP at T0, mmHg  Time from triage to antibiotics, mins | 47.9% (227)  62.4 (19.1)  2.2 [1.4-3.4]  16.3 (6.4)  94.5 (13.2)  72 [40-131] | 50% (105)  62.4 (19)  1.3 [1.1-1.6]  14.2 (6.4)  96.9 (13.3)  88 [46-155] | 46.2% (122)  63 (18)  3.2 [2.5-4.6]  18.0 (5.9  92.7 (12.9)  64 [34-118] | 0.41  0.80  <0.001  <0.001  0.001  0.002 |
| *Fluid volume administered, mL*  Pre-T0,  Between T0-T6  Between T6-T24  Total: preT0-T24 | 1000 [1000-1500]  1927 (1206)  1000 [250-2000]  4567 (1986) | 1000 [1000-1500]  1674 (1110)  1000 [0-2000]  4103 (1909) | 1000 [1000-1900]  2126 (1244)  1165 [540-2000]  4936 (1973) | 0.008  <0.001  0.007  <0.001 |
| Total fluid volume administered prior to starting vasopressors, mL | *N=114*  2000 [1500-3000] | *N=29*  2000 [1500-3000] | *N=85*  2000 [1500-3000] | 0.81 |
| Vasopressor infusion started in ED, n (%)  Vasopressor infusion started before T24, n (%)  Duration of vasopressor infusion, hrs  Time to start vasopressor infusion from T0, hrs | 113 (22.8%)  150 (31.8%)  27.9 [13-51]  2.1 [0.7-4.7] | 29 (13.8%)  36 (17.2%)  21.1 [8-42]  3.2 [1.0-5.6] | 84 (31.8%)  114 (43.3%)  29.8 [15-59]  1.9 [0.7-4.5] | <0.001  <0.001  0.14  0.09 |
| *ICU Outcomes*  Admitted to ICU within 24 hours, n (%) Patients receiving invasive ventilation, n (%) Duration of ventilation, days Patients receiving RRT, n (%)  Duration of RRT, days | 185 (39.2%)  31 (16.9%)  5.6 [2.0-7.3]  13 (7.2%) | 53 (25.5%)  4 (7.5%)  6.0 [4.9-25]  5 (9.4%) | 132 (50%)  27 (20.8%)  5.5 [1.9-7.3]  8 (6.3%) | <0.001  0.031  0.41  0.45 |
| ICU mortality, n (%)  Hospital Mortality, n (%)  Hospital LOS, days | 8.9% (16/179)  7.1% (33/463)  5.4 [2.9-11] | 7.7% (4/52)  5.8% (12/207)  4.2 [2.5-8.2] | 9.4% (12/127)  8.2% (21/256)  6.4 [3.6-14.7] | 0.71  0.32  <0.001 |

T0= time when all 3 inclusion criteria were met; APACHE II: Acute Physiology and Chronic Health Evaluation; SBP: Systolic Blood Pressure; ED: emergency department; ICU: intensive care unit; CCU: Coronary Care Unit; RRT: renal replacement therapy; LOS: Length of stay

**Supplemental table E.** Systolic Blood Pressure <90 AND lactate≥2 mmol/L at T0 vs. patients not meeting these criteria

|  | **Overall**  ***N=470*** | ***Other***  ***N=388*** | **SBP<90 AND lactate ≥2**  **N=82** | **p-value** |
| --- | --- | --- | --- | --- |
| Female, n (%)  Age, years  Lactate T0, mmol/L  APACHE II score  SBP at T0, mmHg  Time from triage to antibiotics, mins | 288 (48.1%)  64.0 (18.5)  2.2 [1.4-3.4]  16.3 (6.4)  94.5 (13.2)  72 [40-131] | 186 (47.9%)  63.0 (18.9)  1.8 [1.3-2.9]  15.7 (6.4)  97.2 (12.7)  77 [44-144] | 40 (48.8%)  63.8 (15.7)  3.5 [2.5-4.9]  19.3 (5.7)  81.5 (5.7)  50 [28-94] | 0.89  0.011  <0.001  <0.001  <0.001  <0.001 |
| *Fluid volume administered, mL*  Pre-T0,  Between T0-T6  Between T6-T24  Total: preT0-T24 | 1000 [1000-1500]  1921 (1201)  1000 [250-2000]  4200 [3000-5700] | 1  000 [1000-1500]  1836 (1179)  1000 [125-2000]  4000 [3000-5500] | 1000 [1000-1900]  2317 (1231)  1250 [625-2000]  4805 [3900-6170] | 0.52  0.001  0.29  0.002 |
| Total fluid volume administered prior to starting vasopressors, mL | *N=112*  2000 [1500-3000] | *N=70*  2300 [1832-3000] | *N=82*  2000 [1250-3000] | 0.14 |
| Vasopressor infusion started in ED, n (%)  Vasopressor infusion started before T24, n (%)  Duration of vasopressor infusion, hrs  Time to start vasopressor infusion from T0, hrs | 111 (23.6%)  147 (31.4%)  28 [13-53]  2.1 [0.7-4.8] | 69 (17.8%)  94 (24.4%)  26 [11-51]  2.5 [1.1-5.5] | 42 (51.2%)  53 (64.6%)  32 [19-69]  1.8 [0.4-3.1] | <0.001  <0.001  0.23  0.028 |
| *ICU Outcomes*  Admitted to ICU within 24 hours, n (%) Patients receiving invasive ventilation, n (%) Duration of ventilation, days Patients receiving RRT, n (%)  Duration of RRT, days | 182 (38.9%)  30 (16.7%)  5.7 [2-7.5]  12 (6.7%)  3.1 [0.5-8.8] | 128 (33.2%)  22 (17.3%)  6.0-[2.7-7.1]  9 (7.1%)  2.2 [0.6-11] | 54 (65.9%)  8 (25.1%)  4.4 [1.9-7.7]  3 (5.8%)  4.0 [0.2-6.8] | <0.001  0.72  0.81  0.74  0.71 |
| ICU mortality, n (%)  Hospital Mortality, n (%)  Hospital LOS, days | 15 (8.5%)  7.0% (32/459)  5.4 [3.0-11.1] | 14 (11.2%)  6.8% (26/382)  5.1 [2.9-9.3] | 1 (2%)  6 (7.8%)  8.4 [4.0-15] | 0.046  0.76  0.001 |

T0= time when all 3 inclusion criteria were met; APACHE II: Acute Physiology and Chronic Health Evaluation; SBP: Systolic Blood Pressure; ED: emergency department; ICU: intensive care unit; CCU: Coronary Care Unit; RRT: renal replacement therapy; LOS: Length of stay

**Supplemental table F.** Abdominal source of sepsis vs other source

T0= time when all 3 inclusion criteria were met; APACHE II: Acute Physiology and Chronic Health Evaluation; SBP: Systolic Blood Pressure; ED: emergency department; ICU: intensive care unit; CCU: Coronary Care Unit; RRT: renal replacement therapy; LOS: Length of stay

|  | **Overall**  ***N=588*** | ***other***  ***N=508*** | **abdominal**  **N=80** | **p-value** |
| --- | --- | --- | --- | --- |
| Female, n (%)  Age, years  Lactate T0, mmol/L  APACHE II score  SBP at T0, mmHg  Time from triage to antibiotics, mins | 288 (49%)  62.4 (19.1)  2.2 [1.4-3.4]  15.2 (6.7)  94.7 (13.7)  77 [42-147] | 251 (59.4%)  62.4 (19)  2.1 [1.4-3.3]  15.2 (6.6)  95 (13.5)  75 [40-142] | 37 (46.3%)  63 (18)  2.7 [1.7-4.5]  15.1 (6.8)  93.2 (14.9)  103 [48-200] | 0.60  0.80  0.007  0.81  0.30  0.012 |
| *Fluid volume administered, mL*  Pre-T0,  Between T0-T6  Between T6-T24  Total: preT0-T24 | 1000 [1000-1500]  1908 (1230)  1000 [200-2000]  4521 (1980) | 1000 [1000-1500]  1895 (1227)  1000 [62-2000]  4468 (1959) | 1000 [1000-1754]  1987 (1253)  1350 [800-2028]  4859 (2095) | 0.76  0.54  0.028  0.10 |
| Total fluid volume administered prior to starting vasopressors, mL | *N=134*  2000 [1500-3000] | *N=108*  2000 [1500-3000] | *N=26*  2000 [1200-4000] | 0.98 |
| Vasopressor infusion started in ED, n (%)  Vasopressor infusion started before T24, n (%)  Duration of vasopressor infusion, hrs  Time to start vasopressor infusion from T0, hrs | 133 (22.6%)  172 (29.7%)  27 [12-48]  2.5 [0.8-5.0] | 107 (21.1%)  77 (27.1%)  28 [13-51]  2.6 [1.0-4.8] | 26 (32.5%)  95 (32.1%)  25 [3.8-45]  2.0 [0.4-5.7] | 0.023  0.19  0.23  0.45 |
| *ICU Outcomes*  Admitted to ICU within 24 hours, n (%) Patients receiving invasive ventilation, n (%) Duration of ventilation, days Patients receiving RRT, n (%)  Duration of RRT, days | 218 (37.2%)  36 (16.7%)  5.6 [0.6-6.8]  13 (6.1%)  2.2 [0.6-6.8] | 185 (36.5%)  28 (15.4%)  5.8 [1.8-7.8]  10 (5.6%)  1.7 [0.6-11] | 33 (41.8%)  8 (24.2%)  4.8 [2.7-5.8]  3 (9.1%)  4.0 [0.5-6.8] | 0.37  0.21  0.52  0.44  1.00 |
| ICU mortality, n (%)  Hospital Mortality, n (%)  Hospital LOS, days | 18 (8.6%)  36 (6.2%)  5.1 [2.8-10] | 13 (7.3%)  30 (6%)  5.0 [2.7-9.8] | 5 (15.2%)  6 (7.6%)  6.0 [3.8-14] | 0.14  0.59  0.06 |

|  | **Overall**  ***N=588*** | ***other***  ***N=390*** | **Resp Source**  **N=198** | **p-value** |
| --- | --- | --- | --- | --- |
| Female, n (%)  Age, years  Lactate T0, mmol/L  APACHE II score  SBP at T0, mmHg  Time from triage to antibiotics, mins | 288 (49%)  62.5 (19.1)  2.2 [1.4-3.4]  15.2 (6.7)  94.7 (13.7)  77 [42-147] | 198 (50.8%)  61.2 (19.8)  2.3 [1.4-3.5]  14.9 (6.9)  94.4 (13.8)  93 [47-160] | 90 (45.5%)  64.8 (17.4)  2 [1.5-3.2]  15.8 (6.2)  95.4 (13.7)  61 [30-119] | 0.22  0.033  0.19  0.16  0.37  <0.001 |
| *Fluid volume administered, mL*  Pre-T0,  Between T0-T6  Between T6-T24  Total: preT0-T24 | 1000 [1000-1500]  1789 [1000-2500]  1000 [200-2000]  4521 (1980) | 1000 [1000-1500]  2000 [1000-2900]  200 [350-2000]  4741 (1977) | 1000 [1000-1500]  1500 [1000-2400]  1000 [0-1614]  4087 (1919) | 0.18  0.005  0.001  <0.001 |
| Total fluid volume administered prior to starting vasopressors, mL | *N=134*  2000 [1500-3000] | *N=95*  2184 [1500-3100] | *N=39*  2000 [1500-3000] | 0.32 |
| Vasopressor infusion started in ED, n (%)  Vasopressor infusion started before T24, n (%)  Duration of vasopressor infusion, hrs  Time to start vasopressor infusion from T0, hrs | 133 (22.6%)  177 (30.2%)  26.6 [12-48]  2.5 [0.8-5.0] | 94 (24.1%)  127 (32.7%)  28 [12-48]  2.5 [1.0-5.3] | 39 (19.7%)  95 (25.3%)  26 [12-54]  2.1 [0.7-4.7] | 0.23  0.06  0.89  0.55 |
| *ICU Outcomes*  Admitted to ICU within 24 hours, n (%) Patients receiving invasive ventilation, n (%) Duration of ventilation, days Patients receiving RRT, n (%)  Duration of RRT, days | 218 (37.2%)  36 (16.7%)  5.6 [2.0-7.1]  13 (6.1%)  2.2 [0.6-6.8] | 141 (36.3%)  21 (15.2%)  4.8 [2.4-6.4]  9 (6.6%)  4.0 [0.9-6.8] | 77 (38.9%)  15 (19.5%)  6.0 [1.8-7.8]  4 (5.3%)  1.2 {0.2-6.6] | 0.55  0.42  0.81  0.70  0.32 |
| ICU mortality, n (%)  Hospital Mortality, n (%)  Hospital LOS, days | 18 (8.6%)  36 (6.2%)  5.1 [2.8-10] | 13 (9.5%)  25 (6.5%)  4.9 [2.8-10.1] | 5 (6.8%)  11 (5.7%)  5.6 [2.9-10.1] | 0.52  0.69  0.32 |

**Supplemental table G.** Respiratory source of sepsis vs other source

T0= time when all 3 inclusion criteria were met; APACHE II: Acute Physiology and Chronic Health Evaluation; SBP: Systolic Blood Pressure; ED: emergency department; ICU: intensive care unit; CCU: Coronary Care Unit; RRT: renal replacement therapy; LOS: Length of stay

**Supplemental table H.** Age <65 vs ≥65 years

|  | **Overall**  ***N=591*** | ***Age <65***  ***N=286*** | **Age ≥65**  **N=297** | **p-value** |
| --- | --- | --- | --- | --- |
| Female, n (%)  Age, years  Lactate T0, mmol/L  APACHE II score  SBP at T0, mmHg  Time from triage to antibiotics, mins | 290 (49.2%)  62.4 (19.1)  2.2 [1.4-3.4]  15.2 (6.7)  94 [87-100]  77 [42-147] | 164 (57.5%)  46.7 (13.6)  1.9 [1.2-2.9]  11.6 (6.2)  94 [89-101]  93 [47-172] | 122 (41.1%)  77.7 (8.0)  2.4 [1.6-3.7]  18.7 (5.1)  93 [85-100]  70 [39-135] | <0.001  <0.001  <0.001  <0.001  0.10  0.011 |
| *Fluid volume administered, mL*  Pre-T0,  Between T0-T6  Between T6-T24  Total: preT0-T24 | 1000 [1000-1500]  1896 (1217)  1000 [225-2000]  4509 (1968) | 1000 [1000-1650]  2084 (1309)  1141 [95-2000]  4843 (2101) | 1000 [1000-1500]  1717 (1095)  1000 [250-1898]  4187 (1776) | 0.50  <0.001  0.06  <0.001 |
| Total fluid volume administered prior to starting vasopressors, mL | *N=132*  2000 [1500-3000] | *N=55*  2500 [2000-4000] | *N=77*  2000 [1500-3000] | 0.012 |
| Vasopressor infusion started in ED, n (%)  Vasopressor infusion started before T24, n (%)  Duration of vasopressor infusion, hrs  Time to start vasopressor infusion from T0, hrs | 131 (22.5%)  172 (29.7%)  26.2 (12-48]  2.5 [(0.8-5.0] | 54 (18.9%)  77 (27.1%)  32.4 [13-48]  2.8 [1.1-6.2] | 77 (25.9%)  95 (32.1%)  25.2 [11-46]  2.5 [0.7-4.5] | 0.042  0.19  0.53  0.16 |
| *ICU Outcomes*  Admitted to ICU within 24 hours, n (%) Patients receiving invasive ventilation, n (%) Duration of ventilation, days Patients receiving RRT, n (%)  Duration of RRT, days | 214 (36.9%)  35 (16.6%)  5.5 [1.9-7.3]  11 (5.3%)  1.3 [0.5-11] | 99 (34.7%)  25 (25.8%)  7.7 [1.8-7.1]  8 (8.2%)  1.1 [0.4-6.6] | 115 (39%)  10 (8.8%)  3.1 [2.7-7.8]  3 (2.7%)  4.1 [0.5-20] | 0.29  0.001  0.87  0.07  0.61 |
| ICU mortality, n (%)  Hospital Mortality, n (%)  Hospital LOS, days | 18 (8.7%)  35 (6.1%)  5.1 [2.8-10] | 7 (7.4%)  13 (4.6%)  4.7 [2.7-9.1] | 11 (9.9%)  22 (7.6%)  5.9 [3-11.4] | 0.52  0.13  0.008 |

T0= time when all 3 inclusion criteria were met; APACHE II: Acute Physiology and Chronic Health Evaluation; SBP: Systolic Blood Pressure; ED: emergency department; ICU: intensive care unit; CCU: Coronary Care Unit; RRT: renal replacement therapy; LOS: Length of stay

**Supplemental table I.** Cardiovascular comorbidities absent or present

T0= time when all 3 inclusion criteria were met; APACHE II: Acute Physiology and Chronic Health Evaluation; SBP: Systolic Blood Pressure; ED: emergency department; ICU: intensive care unit; CCU: Coronary Care Unit; RRT: renal replacement therapy; LOS: Length of stay

|  | **Overall**  ***N=591*** | ***No known CV comorbidities***  ***N=401*** | **Known CV comorbidities**  **N=190** | **p-value** |
| --- | --- | --- | --- | --- |
| Female, n (%)  Age, years  Lactate T0, mmol/L  APACHE II score  SBP at T0, mmHg  Time from triage to antibiotics, mins | 290 (49.2%)  62.4 (19.1)  2.2 [1.4-3.4]  15.2 (6.7)  94 [87-100]  77 [42-148] | 210 (52.5%)  56.9 (19.1)  2.1 [1.3-3.2]  13.1 (6.4)  94 [88-101]  84 [45-150] | 80 (42.1%)  74.5 (12.1)  2.4 [1.5-3.9]  19.5 (5.0)  92 [85-100]  69 [39-146] | 0.018  <0.001  0.012  <0.001  0.09  0.11 |
| *Fluid volume administered, mL*  Pre-T0,  Between T0-T6  Between T6-T24  Total: preT0-T24 | 1000 [1000-1500]  1908 (1230)  1000 [200-2000]  4518 (1980) | 1000 [1000-1500]  1936 (1232)  1100 [250-2000]  4631 (2007) | 1000 [1000-1500]  1849 (1227)  1000 [62-1765]  4278 (1905) | 0.25  0.43  0.028  0.043 |
| Total fluid volume administered prior to starting vasopressors, mL | *N=135*  2000 [1500-3000] | N=84  2500 [2000-3500] | *N=51*  2000 [1125-2750] | 0.001 |
| Vasopressor infusion started in ED, n (%)  Vasopressor infusion started before T24, n (%)  Duration of vasopressor infusion, hrs  Time to start vasopressor infusion from T0, hrs | 134 (22.7%)  177 (30.2%)  26.6 [12-48]  2.5 [0.8-5.0] | 83 (20.8%)  104 (26.1%)  33.2 [11-51]  2.6 [20.9-5.5] | 51 (26.8%)  73 (38.6%)  25 [13-44]  2.4 [0.7-4.7] | 0.10  0.002  0.89  0.86 |
| *ICU Outcomes*  Admitted to ICU within 24 hours, n (%) Patients receiving invasive ventilation, n (%) Duration of ventilation, days Patients receiving RRT, n (%)  Duration of RRT, days | 218 (37.1%)  36 (16.7%)  5.6 [2.0-7.1]  13 (6.1%)  2.2 [0.6-6.8] | 135 (33.9%)  26 (19.5%)  5.7 [2.0-7.1]  9 (6.8%)  2.2 [0.6-6.8] | 83 (43.9%)  10 (12.2%)  5.5 [1.8-8.4]  4 (4.9%)  2.6 [0.9-7.5] | 0.0019  0.16  0.98  0.58  1.00 |
| ICU mortality, n (%)  Hospital Mortality, n (%)  Hospital LOS, days | 18 (8.6%)  36 (6.2%)  5.1 [2.8-10] | 7 (5.3%)  15 (3.8%)  4.8 [2.7-9.1] | 11 (13.9%)  21 (11.4%)  6.2 [3.3-13] | 0.031  <0.001  0.005 |
