# Supplementary material for: The Australasian Resuscitation In Sepsis Evaluation: Fluids or vasopressors in emergency department sepsis (ARISE FLUIDS), a multi‐centre observational study describing current practice in Australia and New Zealand
Source: Emerg Med Australas. 2020 Feb 10;32(4):586–98. doi: 10.1111/1742-6723.13469 (PMC7496107; doi:10.1111/1742-6723.13469)
Supplement: Supplementary file 2 — Table S10. Severity of illness – by APACHE II quartiles at T0. Table S11. Hospital type. [file EMM-32-586-s002.docx]

**Supplemental table J.** Severity of illness – by APACHE II quartiles at T0

|  | **Overall**  ***N=590*** | ***0-10***  ***N=158*** | **11-16**  **N=180** | **17-20**  ***N=130*** | **>21**  **N=122** |
| --- | --- | --- | --- | --- | --- |
| Female, n (%)  Age, years  Lactate T0, mmol/L  APACHE II score  SBP at T0, mmHg  Time from triage to antibiotics, mins | 290 (49.2%)  62.4 (19.1)  2.2 [1.4-3.4]  15.2 (6.7)  94.7 (13.7)  77 [42-148] | 100 (63.3%)  44.9 (16.8)  1.4 [1-2.1]  6.7 (2.7)  98 (12.1)  110 [61-210] | 81 (45%)  63.2 (16.6)  2.3 [1.4-3.2]  14 (1.6)  95.8 (14.5)  77 [41-154] | 59 (45.4%)  73 (13.2)  2.3 [1.5-3.5]  18.5 (1.2)  93.7 (12.9)  73 [42-147 | 50 (41%)  72.7 (13.5)  2.9 [1.9-4.7]  24.3 (3.2)  90 (13.9)  52 [30-98] |
| *Fluid volume administered, mL*  Pre-T0,  Between T0-T6  Between T6-T24  Total: preT0-T24 | 1000 [1000-1500]  1908 (1230)  1000 [200-2000]  4518 (1980) | 1000 [1000-1600]  1746 (1257)  1113 [0-2000]  4448 (2044) | 1000 [1000-2000]  1912 (1188)  1000 [310-2000]  4641 (2064) | 1000 [1000-1500]  1846 (1137)  1000 [62-1735]  4161 (1639) | 1000 [1000-1500]  2173 (1319)  1050 [332-2000]  4806 (2061) |
| Total fluid volume administered prior to starting vasopressors, mL | *N=135*  2000 [1500-3000] | N=17  3500 [2668-4100] | *N=43*  2500 [2000-3125] | *N=28*  2000 [1113-2621] | *N=46*  2000 [1500-2750] |
| Vasopressor infusion started in ED, n (%)  Vasopressor infusion started before T24, n (%)  Duration of vasopressor infusion, hrs  Time to start vasopressor infusion from T0, hrs | 134 (22.7%)  177 (30.2%)  27 [12-48]  2.5 [0.8-5.0] | 17 (10.8%)  21 (13.4%)  19 [8-43]  4.5 [3.2-7.3] | 43 (23.9%)  54 (30%)  30 [7-48]  2.0 [0.8-5.5] | 28 (21.5%)  43 (33.3%)  33 [17-48]  2.3 [1.1-4.2] | 46 (37.7%)  59 (48.8%)  26 [13-53]  2.1 [0.7-4.5] |
| *ICU Outcomes*  Admitted to ICU within 24 hours, n (%) Patients receiving invasive ventilation, n (%) Duration of ventilation, days Patients receiving RRT, n (%)  Duration of RRT, days | 218 (37.1%)  36 (16.7%)  5.6 [2.0-7.1]  13 (6.1%)  2.2 [0.6-6.8] | 31 (19.7%)  4 (12.9%)  5.8 [4.2-44]  1 (3.2%)  23 [23-23] | 65 (36.1%)  14 (21.5%)  5.2 [1.8-7.5]  4 (6.2%)  1.7 [0.7-4.5] | 54 (41.9%)  5 (9.4%)  2.2 [1.4-4.4]  2 (3.8%)  0.7 [0.5-0.9] | 68 (56.2%)  13 (19.7%)  5.6 [2.4-10]  6 (9.4%)  4.0 [0.6-11] |
| ICU mortality, n (%)  Hospital Mortality, n (%)  Hospital LOS, days | 18 (8.6%)  36 (6.2%)  5.1 [2.8-10] | 1 (3.2%)  3 (1.9%)  3.9 [2.2-6.8] | 3 (4.7%)  9 (5%)  5.2 [2.8-9.4] | 3 (5.9%)  7 (5.5%)  5.7 [3.1-10] | 11 (17.2%)  17 (14.8%)  7.3 [3.3-17] |

T0= time when all 3 inclusion criteria were met; APACHE II: Acute Physiology and Chronic Health Evaluation; SBP: Systolic Blood Pressure, ED: emergency department; ICU: intensive care unit; CCU: Coronary Care Unit; RRT: renal replacement therapy; LOS: Length of stay

**Supplemental table K.** Hospital Type

|  | **Overall**  ***N=591*** | ***Metropolitan / District***  ***N=129*** | **Private**  **N=25** | **Rural / Regional**  ***N=117*** | **Tertiary**  **N=320** |
| --- | --- | --- | --- | --- | --- |
| Female, n (%)  Age, years  Lactate T0, mmol/L  APACHE II score  SBP at T0, mmHg  Time from triage to antibiotics, mins | 290 (49.2%)  62.4 (19.1)  2.2 [1.4-3.4]  15.2 (6.7)  94.7 (13.7)  77 [42-148] | 74 (57.4%)  63.4 (20)  1.9 [1.3-3.4]  14.2 (6.5)  96.5 (15.7)  95 [55-177] | 11 (44%)  67.1 (19.6)  1.8 [1.5-3.3]  14.6 (5.8)  88.9 (11.1)  60 [41-98] | 49 (41.9%)  62.3 (17.3)  2.4 [1.7-3.6]  16.1 (7.0)  94.7 (11.8)  74 [39-127] | 156 (48.9%)  61.8 (19.3)  2.2 [1.3-3.4]  15.3 (6.7)  94.5 (13.6)  75 [40-154] |
| *Fluid volume administered, mL*  Pre-T0,  Between T0-T6  Between T6-T24  Total: preT0-T24 | 1000 [1000-1500]  1908 (1230)  1000 [200-2000]  4518 (1980) | 1000 [1000-1650]  1755 (1258)  1000 [0-1969]  4228 (2168) | 1100 [1000-2000]  2579 (1217)  1400 [660-2373]  5273 (1889) | 1000 [1000-1500]  1882 (1093)  1080 [125-2000]  4505 (1916) | 1000 [1000-1500]  1927 (1254)  1000 [250-2000]  4580 (1917) |
| Total fluid volume administered prior to starting vasopressors, mL | *N=135*  2000 [1500-3000] | *N=34*  2000 [1125-3000] | *N=6*  2000 [2000-2500] | *N=28*  2175 [1500-3015] | *N=66*  2000 [1500-3000] |
| Vasopressor infusion started in ED, n (%)  Vasopressor infusion started before T24, n (%)  Duration of vasopressor infusion, hrs  Time to start vasopressor infusion from T0, hrs | 134 (22.7%)  177 (30.2%)  26.6 [12-48]  2.5 [0.8-5.0] | 34 (26.4%)  38 (29.7%)  33 [6.8-46]  2.3 [0.5-5.0] | 6 (24%)  10 (40%)  42 [36-59]  1,7 [0.8-2.8] | 28 (23.9%)  37 (32.2%)  25 [13-48]  2.5 [0.4-5.5] | 66 (20.7%)  92 (28.8%)  25 [11-49]  2.6 [1.2-5.0] |
| *ICU Outcomes*  Admitted to ICU within 24 hours, n (%) Patients receiving invasive ventilation, n (%) Duration of ventilation, days Patients receiving RRT, n (%)  Duration of RRT, days | 218 (37.1%)  36 (16.7%)  5.6 (2.0-7.1]  13 (6.1%)  2.2 [0.6-6.8] | 50 (39.1%)  3 (6%)  5.7 [5.6-6.0]  2 (4.1%)  2.1 [0.2-4.0] | 14 (58.3%)  2 (14.3%)  1.7 [1.4-2.0]  1 (7.1%)  0.2 [0.2-0.2] | 44 (37.9%)  8 (19%)  3.9 [1.8-7.1]  1 (2.4%)  0.6 [0.6-0.6] | 110 (34.5%)  23 (21.1%)  5.7 [2.7-8.4]  9 (8.3%)  4.1 [1.3-11] |
| ICU mortality, n (%)  Hospital Mortality, n (%)  Hospital LOS, days | 18 (8.6%)  36 (6.2%)  5.1 [2.8-10] | 5 (10.6%)  9 (7.1%)  4.7 [2.5-9.2] | 1 (7.1%)  3 (12.5%)  5.3 [2.4-13] | 1 (2.4%)  4 (3.6%)  5.1 [2.9-9.2] | 11 (10.2%)  20 (6.3%)  5.2 [2.9-11] |

T0= time when all 3 inclusion criteria were met; APACHE II: Acute Physiology and Chronic Health Evaluation; SBP: Systolic Blood Pressure, ED: emergency department; ICU: intensive care unit; CCU: Coronary Care Unit; RRT: renal replacement therapy; LOS: Length of stay
